# Supplementary material for: Neuronal Yin Yang1 in the prefrontal cortex regulates transcriptional and behavioral responses to chronic stress in mice
Source: Nat Commun. 2022 Jan 10;13:55. doi: 10.1038/s41467-021-27571-3 (PMC8748737; doi:10.1038/s41467-021-27571-3)
Supplement: Supplementary file 1 — Supplementary Information [file 41467_2021_27571_MOESM1_ESM.pdf]

SUPPLEMENTARY INFORMATION

Neuronal Yin Yang1 in the prefrontal cortex regulates transcriptional and behavioral responses to chronic stress in mice

Deborah Y. Kwon[1,2], Bing Xu[,2,*], Peng Hu[1,2,*], Ying-Tao Zhao[1,2], Jonathan A. Beagan[2,3], Jonathan H. Nofziger[1,2], Yue Cui[1,2], Jennifer E. Phillips-Cremins[2,3], Julie A. Blendy[4], Hao Wu[1,2], Zhaolan Zhou[1,2*]

[1]Department of Genetics,

University of Pennsylvania Perelman School of Medicine,

Philadelphia, PA 19104, USA

[2]Epigenetics Institute,

University of Pennsylvania,

Philadelphia, PA 19104, USA

[3]Department of Bioengineering,

University of Pennsylvania,

Philadelphia, PA 19104, USA

[4]Department of Systems Pharmacology and Translational Therapeutics,

University of Pennsylvania Perelman School of Medicine,

Philadelphia, PA 19104, USA

*These authors contributed equally

*To whom correspondence should be addressed: zhaolan@pennmedicine.upenn.edu

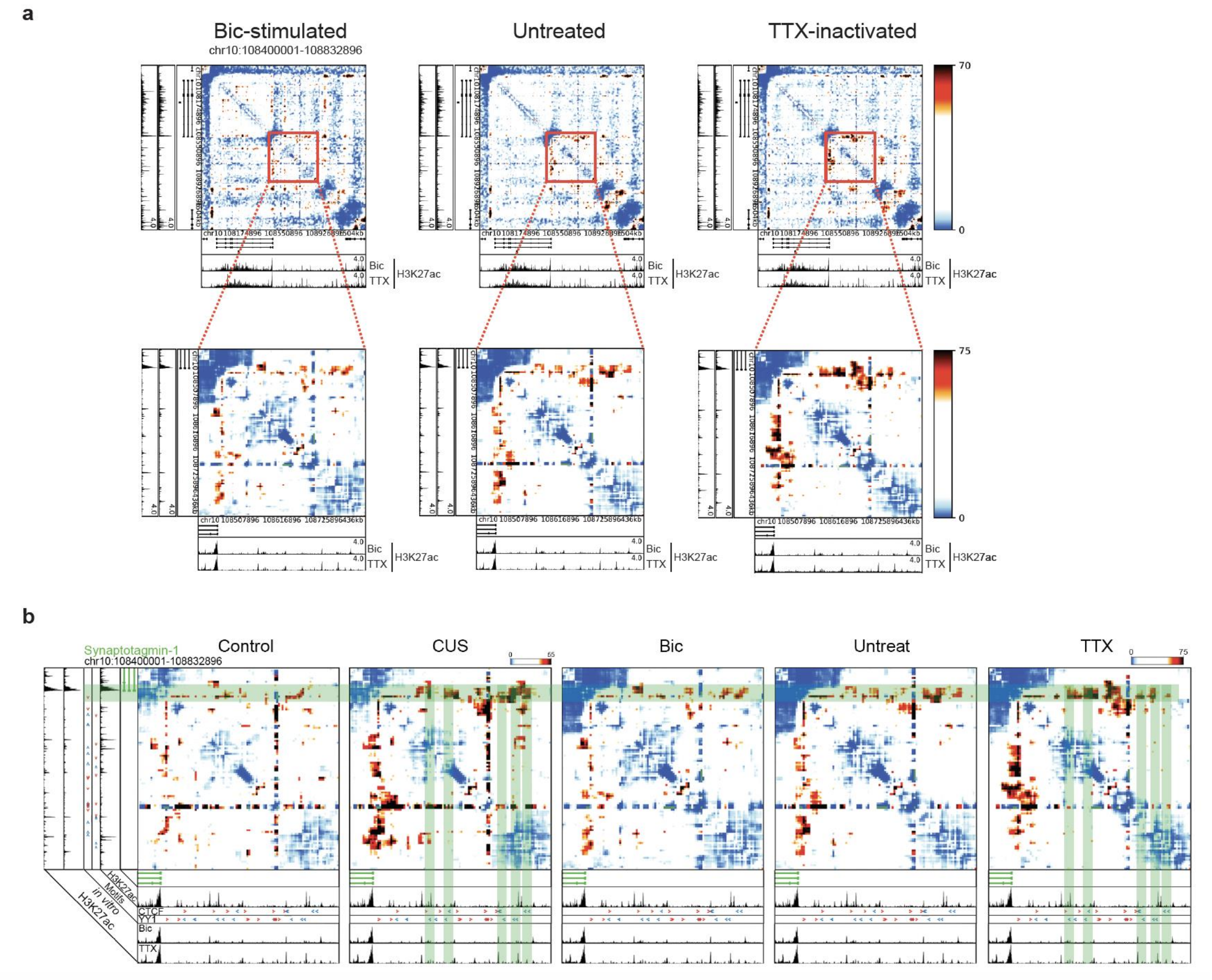

Supplementary Figure 1. Increased chromatin interactions associated with neuronal inactivation at *Syt1* locus. (a) Background-corrected interaction frequency heatmaps depicting chromatin interactions at the *Syt1* gene locus in mouse primary cortical neurons stimulated with bicuculline (Bic), left untreated, or inactivated with tetrodotoxin (TTX). Red box in each heatmap marks location of zoomed-in heatmap below. H3K27ac ChIP enrichment from the Bic and TTX conditions, as well as from adult mouse cortical tissues, are plotted below each heatmap. (b) Heatmaps plotting genomic locations and directionality of CTCF and YY1 binding sequences in regions showing increased chromatin interactions in CUS mice and TTX-treated neuronal cultures (green shaded boxes). Legends depicts background-corrected interaction scores.

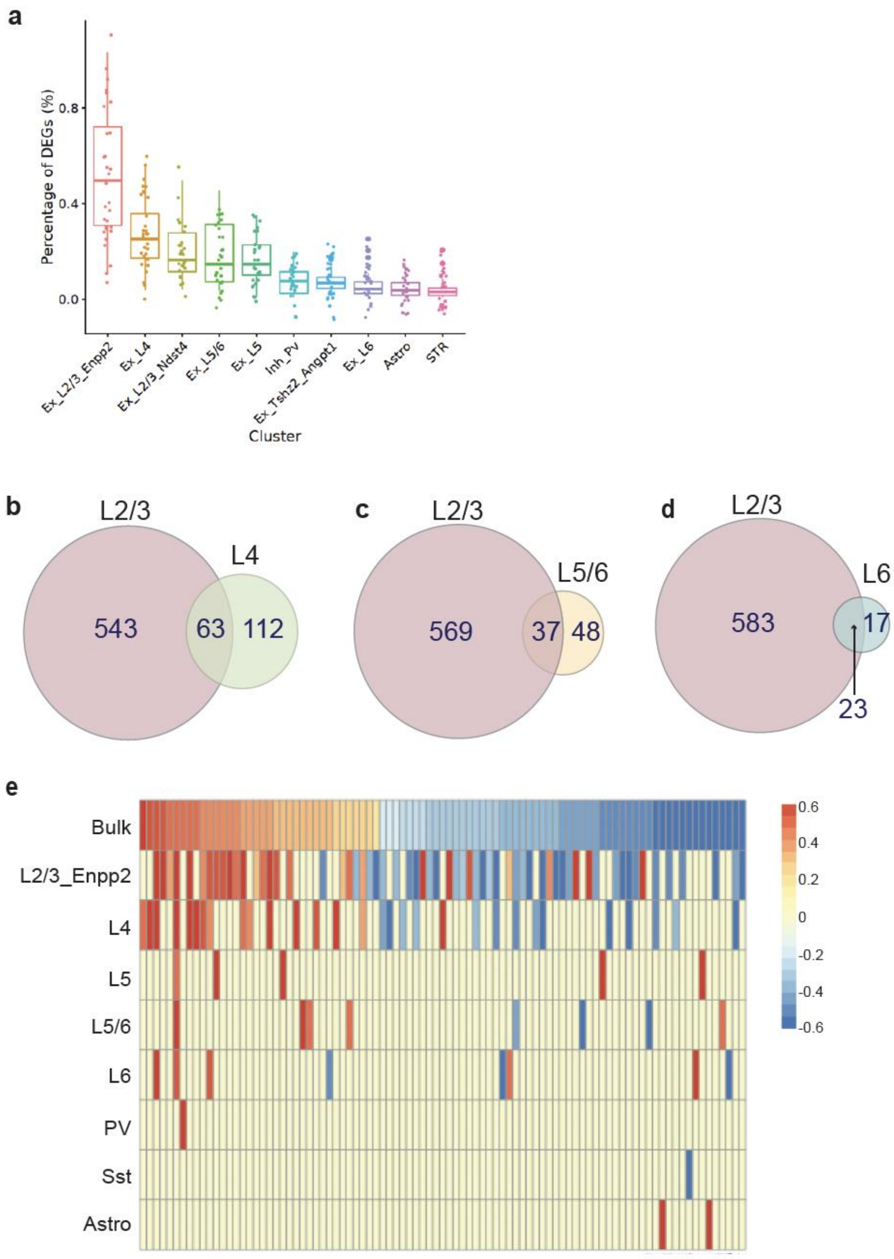

Supplementary Figure 2. CUS precipitates shared and unique transcriptional changes in cortical excitatory neurons. (a) Boxplots illustrating the proportion of DEGs in each cell type after 30 repeated random downsamplings (two-sided Wilcoxon test; P = 6.26e-05). Venn diagrams displaying shared CUS DEGs between L2/3 neurons and (b) L4, (c) L5/6, and (d) L6 neurons. (e) Heatmap of CUS DEGs shared between bulk nuclear RNA-seq and sNucDrop-seq clusters. Legend depicts z-score of normalized gene expression.

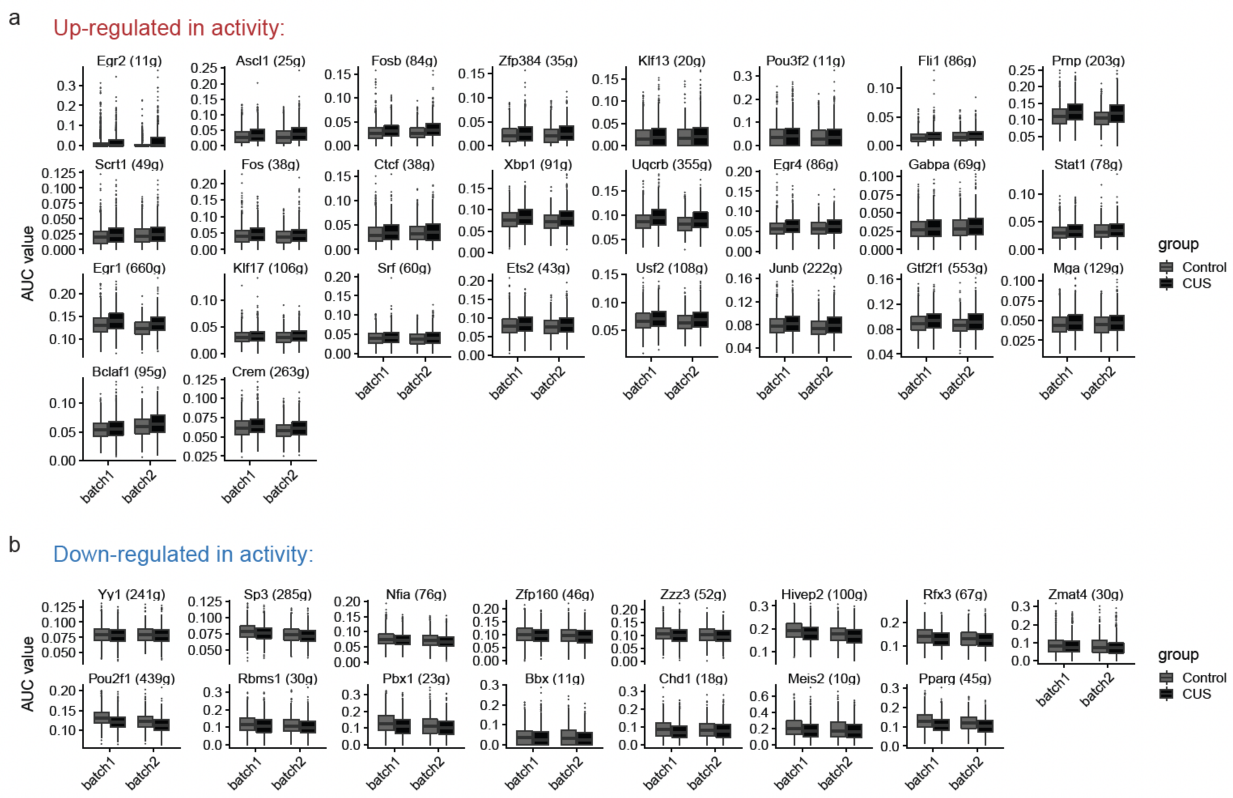

Supplementary Figure 3. CUS deregulates transcription factor activity in L2/3 excitatory neurons. Boxplots of AUC values determined by SCENIC for the transcription factors whose activities were found to be significantly (a) upregulated and (b) downregulated in CUS L2/3 nuclei compared to controls. Samples are shown separated by sequencing batch (n=4 per group). Boxes display the median (center line) and interquartile range (from the 25th to 75th percentile), the whiskers represent 1.5 times the interquartile range and the circles represent outliers.

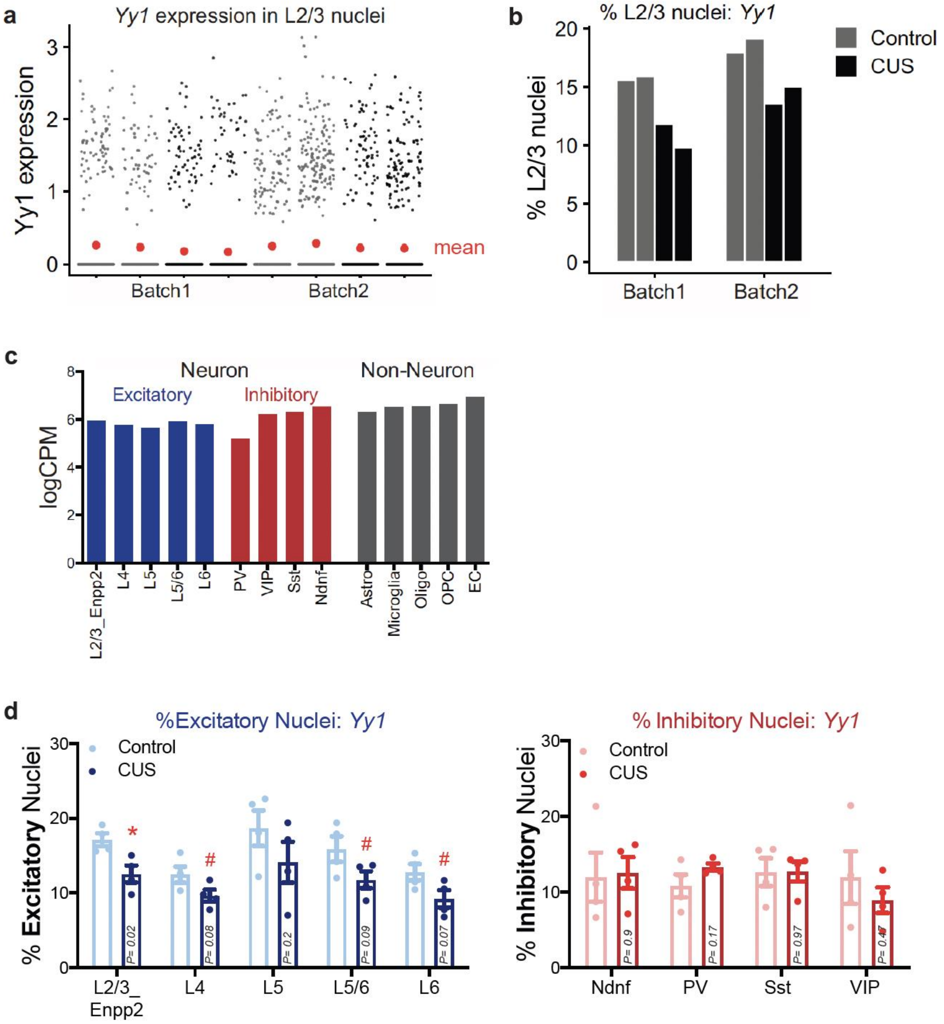

Supplementary Figure 4. CUS decreases *Yy1* expression in cortical excitatory neurons. (a) Mean expression of *Yy1* (ln(TPM100K+1)) is decreased in CUS L2/3 nuclei compared to their respective controls for each sequencing batch. Red dots represent mean *Yy1* expression. (b) The percentage of L2/3 neuronal nuclei expressing *Yy1* (black bars) is decreased in CUS samples (black bars) versus controls (grey bars) in both sequencing batches. (c) Transcript levels (logCPM) of *Yy1* across all major subtypes of excitatory (blue), inhibitory (red), and non-neuronal (grey) cells. (d) Trend towards decreased percent of CUS nuclei expressing *Yy1* is seen across all major cortical excitatory neuronal subtypes but not in subtypes of inhibitory nuclei (n=4 per group). P values for each neuronal subtype is included in the CUS nuclei bar. #P<0.1; *P<0.05. Error bars represent s.e.m. Source data are provided as a Source Data file below.

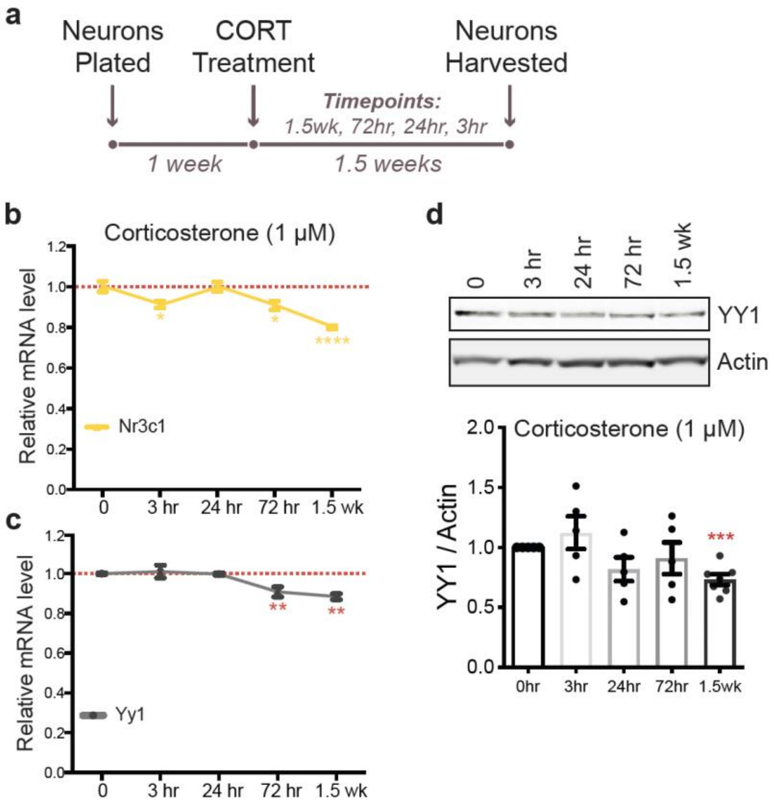

Supplementary Figure 5. Chronic corticosterone (CORT) treatment decreases *Yy1* transcript and protein levels in mouse primary cortical neurons. (a) Timeline of CORT treatment in mouse primary neuronal cell culture. (b) *Nr3c1* transcript levels, as measured by quantitative RT-PCR, are significantly decreased by 3h (P= 0.03), 72h (P= 0.02), and 1.5wk (P<0.0001) administration of CORT (One-way ANOVA with Dunnett's multiple comparison test; n=4 per group). (c) *Yy1* transcript levels are significantly decreased by 72h (P= 0.001) and 1.5wk (P= 0.001) administration of CORT (One-way ANOVA with Dunnett's multiple comparison test; n=4 per group). (d) Representative western blot image of YY1 protein levels in murine primary cortical neurons treated with CORT. β-actin was used as a loading control. Quantitative measurement of YY1 protein expression is shown below, depicting significant decreases in YY1 protein levels after 1.5wk administration of CORT relative to 0h vehicle treated controls (Wilcoxon signed-rank test; n=4 per group; P= 0.0009). *P<0.05; **P<0.01; ***P<0.001. Error bars represent s.e.m. Source data are provided as a Source Data file below.

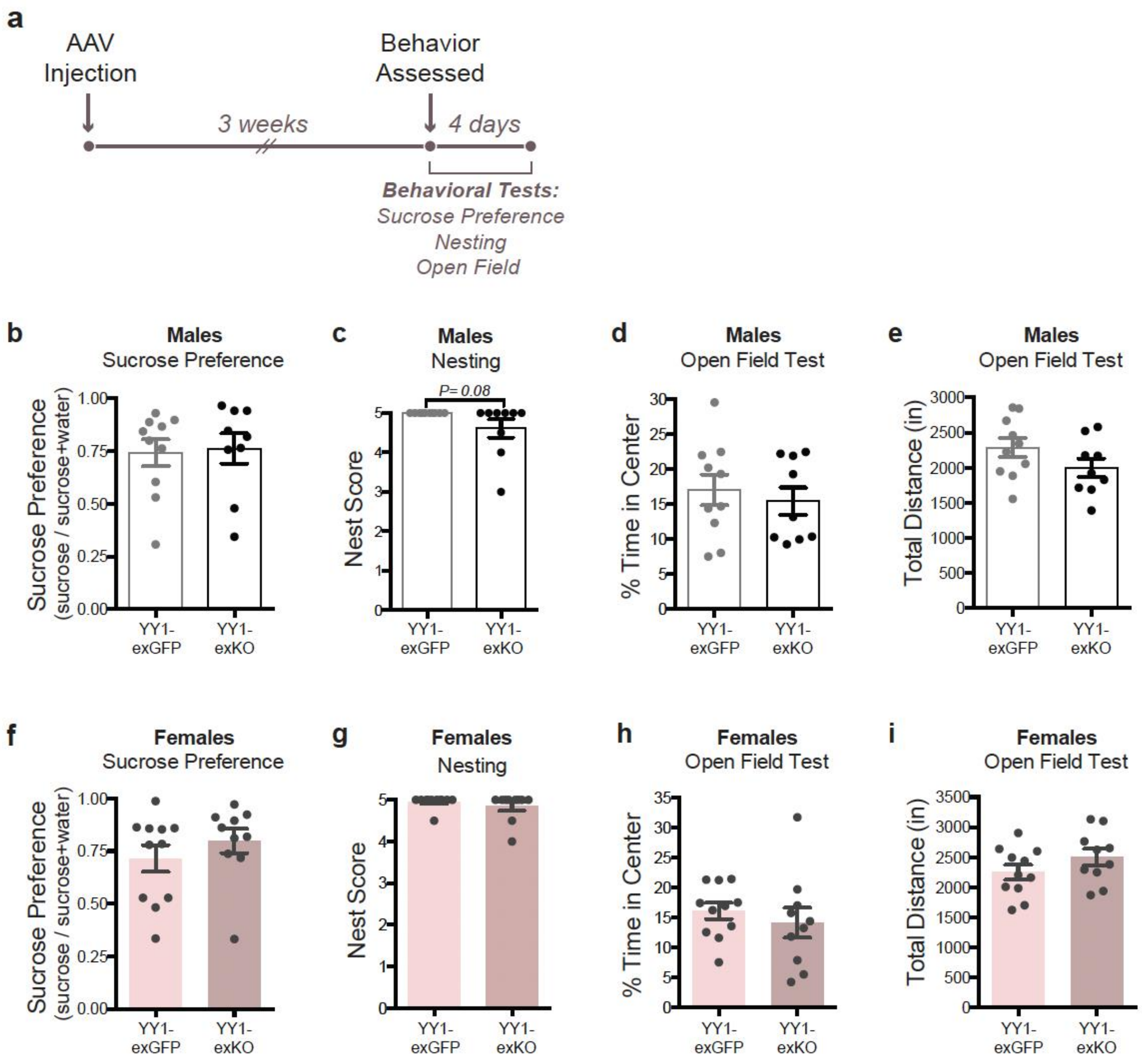

Supplementary Figure 6. Loss of YY1 in PFC excitatory neurons alone does not significantly drive complex behavior in adult male and female mice. (a) Timeline of AAV injections and behavioral testing of mice. (b) Males injected with AAV.CamKII.eGFP-Cre (Yy1-exKO, n=9) show comparable sucrose preference to controls injected with AAV.CamKII.eGFP (Yy1-exGFP, n=10) (Unpaired t-test; P= 0.84). (c) Yy1-exKO males (n=9) show a trend towards decreased nesting behavior relative to Yy1-exGFP (n=10) controls (Mann-Whitney U-test; P= 0.08). (d,e) Loss of YY1 in PFC excitatory neurons alone does not alter (d) exploratory behavior (Mann-Whitney U-test; P= 0.28; n=10 Yy1-exGFP, n=9 Yy1-exKO) and (e) total locomotion in the open field test (Unpaired t-test; P= 0.15; n=10 Yy1-exGFP, n=9 Yy1-exKO). (f-i) Female mice injected with AAV.CamKII.eGFP-Cre (Yy1-exKO) also show comparable (f) sucrose preference (Unpaired t-test, P= 0.34; n=11 Yy1-exGFP, n=10 Yy1-exKO), (g) nesting (Mann-Whitney U-test; P= 0.46, n=11 Yy1-exGFP, n=10 Yy1-exKO), (h) exploratory behavior (Unpaired t-test, P= 0.34; n=11 Yy1-exGFP, n=10 Yy1-exKO), and (i) locomotor activity (Unpaired t-test, P= 0.19; n=11 Yy1-exGFP, n=10 Yy1-exKO) compared to controls injected with AAV.CamKII.eGFP (Yy1-exGFP). Error bars represent s.e.m. and statistical tests were two-sided unless stated otherwise. Source data are provided as a Source Data file below.

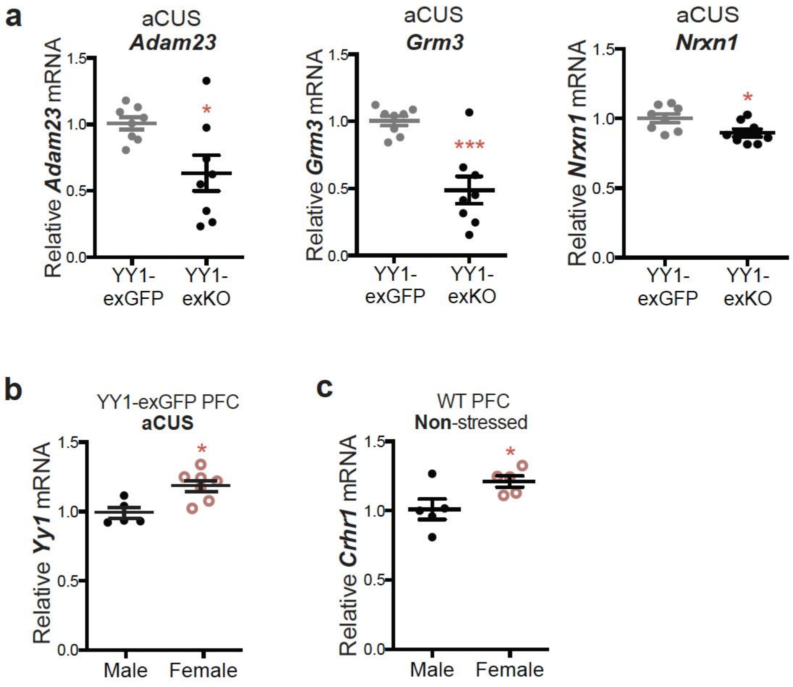

Supplementary Figure 7. Deregulation of synaptic CUS DEGs are seen in medial PFC tissues of aCUS-exposed Yy1-KO mice. (a) Virally infected medial PFC tissues collected from Yy1-exKO males subjected to aCUS show altered expression of the synaptic genes, *Adam23* (Unpaired t-test with Welch's correction; P= 0.02; n=8), *Grm3* (Unpaired t-test with Welch's correction; P= 0.0003; n=8), and *Nrxn1* (Unpaired t-test; P= 0.02) which were also decreased by CUS in bulk nuclear RNA-seq and sNucDrop-seq datasets. (b) Medial PFC tissues isolated from Yy1-exGFP female mice show increased *Yy1* expression relative to Yy1-exGFP males (Unpaired t-test; P= 0.01; n=5 Male, n=7 Female). (c) Medial PFC tissues isolated from wild-type female mice show enhanced expression of *Crhr1* relative to wild-type males (Unpaired t-test; P= 0.04; n=5 per group). *P<0.05; ***P<0.001. Error bars represent s.e.m. and statistical tests were two-sided unless stated otherwise. Source data are provided as a Source Data file below.

CUS Schedule			
Day	Morning (8 am)	Afternoon (1 pm)	Overnight (6 pm)
1			Lights on
2	Cage Rotation (1 hr)	Tilt (3 hrs)	Food Deprivation
3	Restraint (1 hr)	Lights Off (3 hrs)	Stroboscope
4	Swim @Room temp (10 min per mouse)	Cold: cages on ice (1 hr)	Wet Bedding
5	Rat Odor (3 hrs)	Restraint, dark (1 hr)	Isolation (no nestlet)
6	Cage Rotation (1 hr)	New Partner (3 hrs)	Static
7	Cold: cages on ice (1 hr)	Cage tilt, dark (3 hrs)	Wet Bedding
8	Swim @18C (8 min per mouse)	Rat Odor, strobe (3 hrs)	Cage tilt
9	New Partner (3 hrs)	Restraint, shaker (1 hr)	Lights on
10	Cage tilt, dark, wet bedding (3 hrs)	Cold (1 hr)	Stroboscope
11	Cage Rotation (1 hr)	Swim @RT (10 min)	Static
12	Rat odor (3 hrs)	Restraint, dark (1 hr)	Isolation for Behavioral Testing (SPT/Nesting/Food)

Supplementary Table 1. CUS schedule. Schedule of CUS stressors over the 12-day CUS period.

aCUS Schedule			
**Day**	**Morning (8 am)**	**Afternoon (1 pm)**	**Overnight (6 pm)**
1			Lights on, Wet Bedding
2	Cage Rotation (1 hr)	Tilt (3 hrs)	Static
3	Restraint (1 hr)	Lights Off (3 hrs)	Stroboscope
4	Swim @RT (10 min per mouse)	Cold: cages on ice (1 hr)	Isolation (for SPT, Nesting)

Supplementary Table 2. aCUS Schedule. Schedule of aCUS stressors over the 3-day CUS period.
