# Supplementary material for: Neuronal Yin Yang1 in the prefrontal cortex regulates transcriptional and behavioral responses to chronic stress in mice
Source: Nat Commun. 2022 Jan 10;13:55. doi: 10.1038/s41467-021-27571-3 (PMC8748737; doi:10.1038/s41467-021-27571-3)
Supplement: Supplementary file 3 — Description of Additional Supplementary Files [file 41467_2021_27571_MOESM3_ESM.docx]

**Description of Additional Supplementary Files**

File Name: Supplementary Data 1
Description:
List of 1,362 differentially expressed transcripts identified via bulk nuclear RNA-Seq from 9 individual adult male mice (4 unstressed controls, 5 CUS, FDR <0.05), 832 of which are downregulated and 530 are upregulated.

File Name: Supplementary Data 2
Description:

List of cortical cell type (cluster)-specific CUS-associated differentially expressed genes (DEGs) identified via sNucDrop-seq (2 unstressed control and 2 CUS mice in each batch of sNucDrop-seq for a total of 2 batches, FDR <0.2). Note, the presence of CUS-associated DEGs across virtually every cortical cluster. DEGs in YY1 GRN/regulon are noted in the last column.

File Name: Supplementary Data 3
Description:

Number of cell type (cluster)-specific DEGs identified using the same statistical criteria before and after down-sampling the number of cells in each cluster. Note, L2/3_Enpp2 neurons contain the greatest number of DEGs with and without down-sampling, and that other cortical excitatory neuronal subtypes, L5/6, L5, L6, L2/3_Ndst4, also show more transcriptional deregulation than the non-excitatory neuronal groups analyzed.
