# Supplementary material for: Neuronal Yin Yang1 in the prefrontal cortex regulates transcriptional and behavioral responses to chronic stress in mice
Source: Nat Commun. 2022 Jan 10;13:55. doi: 10.1038/s41467-021-27571-3 (PMC8748737; doi:10.1038/s41467-021-27571-3)
Supplement: Supplementary file 7 — Reporting Summary [file 41467_2021_27571_MOESM7_ESM.pdf]

nature research

Corresponding author(s): Zhaolan Zhou

Last updated by author(s): 2021/11/19

Reporting Summary

Nature Research wishes to improve the reproducibility of the work that we publish. This form provides structure for consistency and transparency in reporting. For further information on Nature Research policies, see our Editorial Policies and the Editorial Policy Checklist.

Statistics

For all statistical analyses, confirm that the following items are present in the figure legend, table legend, main text, or Methods section.

n/a	Confirmed	
☐	☒	The exact sample size (n) for each experimental group/condition, given as a discrete number and unit of measurement
☐	☒	A statement on whether measurements were taken from distinct samples or whether the same sample was measured repeatedly
☐	☒	The statistical test(s) used AND whether they are one- or two-sided<br>*Only common tests should be described solely by name; describe more complex techniques in the Methods section.*
☐	☒	A description of all covariates tested
☐	☒	A description of any assumptions or corrections, such as tests of normality and adjustment for multiple comparisons
☐	☒	A full description of the statistical parameters including central tendency (e.g. means) or other basic estimates (e.g. regression coefficient) AND variation (e.g. standard deviation) or associated estimates of uncertainty (e.g. confidence intervals)
☐	☒	For null hypothesis testing, the test statistic (e.g. F, t, r) with confidence intervals, effect sizes, degrees of freedom and P value noted<br>*Give P values as exact values whenever suitable.*
☒	☐	For Bayesian analysis, information on the choice of priors and Markov chain Monte Carlo settings
☒	☐	For hierarchical and complex designs, identification of the appropriate level for tests and full reporting of outcomes
☒	☐	Estimates of effect sizes (e.g. Cohen's d, Pearson's r), indicating how they were calculated

Our web collection on statistics for biologists contains articles on many of the points above.

Software and code

Policy information about availability of computer code

Data collection	All behavioral data was acquired using SMART Video Tracking software (3.0) with the exception of the tail suspension test data, which was collected with the BIOSEB BIO-TST5 system with accompanying software (v5).
Data analysis	Behavioral data was analyzed using Prism 8. Sequencing data sets were analyzed with R version 3.5.2.

For manuscripts utilizing custom algorithms or software that are central to the research but not yet described in published literature, software must be made available to editors and reviewers. We strongly encourage code deposition in a community repository (e.g. GitHub). See the Nature Research guidelines for submitting code & software for further information.

Data

Policy information about availability of data

All manuscripts must include a data availability statement. This statement should provide the following information, where applicable:

- Accession codes, unique identifiers, or web links for publicly available datasets
- A list of figures that have associated raw data
- A description of any restrictions on data availability

Sequencing data sets used are readily available from the NCBI Gene Expression Omnibus (GEO) under GEO accession number GSE145970. Source data are provided with this paper containing the raw data used in the charts/graphs in Figures 1, 4, 5, 6, and Supplementary Figures 4, 5, 6, and 7 are provided in the Source Data file.

Field-specific reporting

Please select the one below that is the best fit for your research. If you are not sure, read the appropriate sections before making your selection.

[x] Life sciences [ ] Behavioural & social sciences [ ] Ecological, evolutionary & environmental sciences

For a reference copy of the document with all sections, see nature.com/documents/nr-reporting-summary-flat.pdf

Life sciences study design

All studies must disclose on these points even when the disclosure is negative.

Sample size	Sample sizes were not pre-determined. For behavioral data, we used a similar number of animals for CUS experiments that were used in a previously published study on CUS (Yohn et al., 2017).<br>For sNucDrop-seq experiments, the cell numbers typically used in the field were based on previous studies (Macosko et al, 2015, Cell; Hu et al, 2017, Mol Cell). 31,806 nuclei from 8 samples (4 control and 4 CUS) were kept for downstream analysis.<br>Sample sizes for bulk nuclei RNA-seq and ChIP qPCR experiments were based on a previous study (Zurkirchen et al., Nat. Commun., 2020).<br>The numbers of biological replicates used for differential gene expression and sequencing analysis are in compliance with ENCODE consortium long RNA-seq recommendations (≥2 replicates per condition).
Data exclusions	Data were not excluded.
Replication	All experiments were performed with biological replicates using independent experimental animals. All behavioral data were replicated across 2-3 cohorts. Analysis of behavioral findings were performed and corroborated by two blinded experimenters. 5C findings were successfully replicated across two biological replicates. sNucDrop-seq data were consistently replicated across two technical batches, each batch consisting of 4 control and 4 CUS samples. Bulk nuclei RNA-seq data came from one flowcell consisting of samples from one cohort of 4 control and 5 CUS mice. Select DEGs from RNA-seq analysis were experimentally validated in independent samples by RT-PCR. RNAscope experiments were performed using brain samples from one cohort of 3 control and 3 CUS mice. YY1 ChIP-qPCR data came from one cohort consisting of 4 control and 4 CUS mice.
Randomization	Animals were assigned randomly to control and treatment groups.
Blinding	Investigators were blinded to treatment group during behavioral assessment and data analysis.

Reporting for specific materials, systems and methods

We require information from authors about some types of materials, experimental systems and methods used in many studies. Here, indicate whether each material, system or method listed is relevant to your study. If you are not sure if a list item applies to your research, read the appropriate section before selecting a response.

Materials & experimental systems

n/a	Involved in the study	
[ ]	[x]	Antibodies
[x]	[ ]	Eukaryotic cell lines
[x]	[ ]	Palaeontology and archaeology
[ ]	[x]	Animals and other organisms
[x]	[ ]	Human research participants
[x]	[ ]	Clinical data
[x]	[ ]	Dual use research of concern

Methods

n/a	Involved in the study	
[ ]	[x]	ChIP-seq
[x]	[ ]	Flow cytometry
[x]	[ ]	MRI-based neuroimaging

Antibodies

Antibodies used	Western blotting: mouse anti-β-Actin (Abcam; ab8226; diluted 1:10,000); mouse anti-YY1 (Santa Cruz; H-10, sc-7341; diluted 1:1,000); rabbit anti-YY1 (Cell Signaling; D5D9Z Rabbit mAb #46395; 1:1,000). Secondary antibodies (LI-COR) used were anti-mouse IgG IRDye680LT and incubated at dilutions of 1:10,000.<br>ChIP: anti-YY1(#61779, Active Motif) and IgG (#2729S, CST).
Validation	All YY1 antibodies were further validated by us, by western blotting brain tissues from Yy1 floxed animals expressing CamKII-Cre. Mouse β-Actin antibody was validated by Abcam, which states, "Abcam is leading the way in addressing this with our range of recombinant monoclonal antibodies and knockout edited cell lines for gold-standard validation", and that their Abpromise guarantee covering the use of this antibody in western blotting. Furthermore this antibody has been used in over 2000 publications.

Animals and other organisms

Policy information about studies involving animals; ARRIVE guidelines recommended for reporting animal research

Laboratory animals	Male and female C57BL/6J mice (aged 9-10 weeks for CUS) were used for this study.<br>Yy1fl/fl mice were obtained from M. Atchison and maintained on a C57Bl/6J background. Stereotaxis-assisted intra-PFC injections were performed in Yy1fl/fl mice at 10-14 weeks of age.<br>E18 WT C57/BL6 mouse embryos were used for primary neuronal cultures.
Wild animals	Wild animals were not used in this study.
Field-collected samples	No field-collected samples were used in this study.
Ethics oversight	All animal procedures were performed in accordance with the guide for the care and use of laboratory animals and were approved by the Institutional Animal Care and Use Committee (IACUC) at the University of Pennsylvania.

Note that full information on the approval of the study protocol must also be provided in the manuscript.

ChIP-seq

Data deposition

☐ Confirm that both raw and final processed data have been deposited in a public database such as GEO.

☐ Confirm that you have deposited or provided access to graph files (e.g. BED files) for the called peaks.

Data access links<br>*May remain private before publication.*	*For "Initial submission" or "Revised version" documents, provide reviewer access links. For your "Final submission" document, provide a link to the deposited data.*
Files in database submission	*Provide a list of all files available in the database submission.*
Genome browser session<br>(e.g. UCSC)	*Provide a link to an anonymized genome browser session for "Initial submission" and "Revised version" documents only, to enable peer review. Write "no longer applicable" for "Final submission" documents.*

Methodology

Replicates	*Describe the experimental replicates, specifying number, type and replicate agreement.*
Sequencing depth	*Describe the sequencing depth for each experiment, providing the total number of reads, uniquely mapped reads, length of reads and whether they were paired- or single-end.*
Antibodies	*Describe the antibodies used for the ChIP-seq experiments; as applicable, provide supplier name, catalog number, clone name, and lot number.*
Peak calling parameters	*Specify the command line program and parameters used for read mapping and peak calling, including the ChIP, control and index files used.*
Data quality	*Describe the methods used to ensure data quality in full detail, including how many peaks are at FDR 5% and above 5-fold enrichment.*
Software	*Describe the software used to collect and analyze the ChIP-seq data. For custom code that has been deposited into a community repository, provide accession details.*
